# Paediatric CSF acylcarnitine reference ranges

**DOI:** 10.3389/fmolb.2025.1643667

**Published:** 2025-10-01

**Authors:** Ontefetse Neo Plaatjie, A. Marceline Tutu Van Furth, Regan Solomons, Martijn Van Der Kuip, Shayne Mason

**Affiliations:** ^1^ Department of Biochemistry, Biomedical and Molecular Metabolism Research, Faculty of Natural and Agricultural Sciences, North-West University, Potchefstroom, South Africa; ^2^ Department of Paediatric Infectious Diseases and Immunology, Amsterdam University Medical Center, Emma Children’s Hospital, Amsterdam, Netherlands; ^3^ Department of Paediatrics and Child Health, Faculty of Medicine and Health Sciences, Stellenbosch University, Cape Town, South Africa

**Keywords:** acylcarnitines, cerebrospinal fluid (CSF), paediatrics, children, reference ranges

## Abstract

Acylcarnitines play a crucial role in energy metabolism pathways, primarily known for their involvement in the beta oxidation of fatty acids. However, their roles extend beyond mitochondrial transport; they also contribute to the synthesis of lipids in the brain. The alteration of both plasma and cerebrospinal fluid (CSF) acylcarnitine levels has been reported in various central nervous system (CNS) disorders. However, existing CSF acylcarnitine analysis has primarily focused on adults, highlighting a critical gap in paediatrics, and plasma may not fully reflect CNS-specific metabolic changes. This study aimed to establish reference ranges of CSF acylcarnitine concentrations in paediatric patients. Using LC-MS/MS, we profiled CSF acylcarnitines in 57 non-meningitis children. The acylcarnitine concentrations reported in this study range from 0.01 µM to 4.21 µM. These findings provide a critical reference for future research exploring the role of acylcarnitines in paediatric CNS disorders, bridging a significant gap in the current understanding of acylcarnitine metabolism in children.

## Introduction

The acylcarnitine profile has proven to be a valuable tool for detecting and monitoring both acquired and inherited metabolic disorders, particularly in neonates, and continues to be used in identifying inborn errors of metabolism (El-et al., 2014; Jones et al., 2010). L-carnitine is widely known for its role in mitochondrial fatty acid oxidation, primarily facilitating the transport of long-chain fatty acids into the mitochondria for beta oxidation by forming acylcarnitines (conjugates of carnitine and fatty acids). Recent research has reported alterations in acylcarnitines in neurodegenerative disorders, including Alzheimer’s disease, Parkinson’s disease, and multiple sclerosis (Burté et al., 2017; Cristofano et al., 2016; Kasakin et al., 2020; Saiki et al., 2017), primarily in serum and plasma. Changes in cerebrospinal fluid (CSF) carnitines have been observed in neuroinfectious conditions (Plaatjie et al., 2024), suggesting a potential disruption in fatty acid metabolism across various distinct neurological conditions.

Acylcarnitine analysis holds significant potential for improving our understanding of the metabolic alterations underlying brain diseases. This is particularly relevant when analysed in the CSF because this biofluid is in direct contact with the brain and provides a more immediate reflection of metabolic alterations within the central nervous system (CNS). However, research on acylcarnitine profiling in CSF remains limited due to the health and ethical challenges in obtaining this biofluid, particularly in the paediatric patient population. The few existing studies identifying altered carnitines in CSF are predominantly conducted in adult populations. Given that acylcarnitine profiling is widely used to detect inborn errors of metabolism (Gucciardi et al., 2015). CSF acylcarnitine analysis could provide complementary insights into brain-specific metabolic processes. While some studies have reported altered carnitine levels in paediatric CSF, the only well-documented study specifically investigating CSF carnitine levels in children was published in 1998, focusing on children with meningitis and neurological disorders (Shinawi et al., 1998). Since then, research in this area has been limited. This study aims to provide baseline concentration ranges of CSF acylcarnitines in paediatric patients (≤12 years old) that will serve as a foundation for future research exploring the role of acylcarnitines in paediatric neurological disorders.

## Methods

### Patient selection and clinical characteristics

Cerebrospinal fluid samples were retrospectively collected from a historical cohort of paediatric patients between 2009 and 2018. The paediatric sample group used in this investigation reside in an area endemic for TB–the Western Cape province of South Africa. Each participant was referred from local regional clinics to the paediatric service at the Tygerberg Academic Hospital in Cape Town, based on clinical signs and symptoms of meningitis. Upon admission to the hospital, each patient was assessed by the paediatric neurology team, and, when they were stable enough, a CSF sample was collected via lumbar puncture for routine differential diagnostic purposes. Cerebrospinal fluid was collected from a total of 195 participants: 88 controls, 40 cases of viral meningitis (VM), and 67 cases of tuberculous meningitis (TBM). The control group consists of patients who were suspected of having meningitis based on clinical symptoms but proved to be negative for meningitis upon final diagnosis. Within the control group, there were two subgroups–controls without any neurological symptoms (“Nonneuro”, n = 40) and controls exhibiting neurological symptoms (“Neuro”, n = 44), such as various forms of seizures, brain injury, and encephalopathy. Both the VM and TBM cases were subdivided into “probable” and “definite” classifications. For VM, a “definite” VM diagnosis (n = 16) was assigned based upon a positive viral PCR test, whereas a “probable” VM diagnosis (n = 24) was given if the PCR test was negative, but the patient presented with clinical symptoms of VM and CSF pleocytosis (>5 leucocytes/mm^3^). For TBM, a “definite” (n = 30) and “probable” (n = 37) TBM diagnosis was assigned based upon a uniform case definition given by Marais et al. (2010).

### Ethics

Written and informed consent and/or assent were obtained for CSF samples for research purposes. This study was approved by the Health Research Ethics Committee (HREC) of Stellenbosch University (ethics approval no. N16/11/142), the Western Cape Department of Health and Wellness, and the HREC of the North-West University, Potchefstroom campus (ethics approval no. NWU-00063-18-A1-05)Reagents and quantification standards.

We used UPLC-grade solvents (water and acetonitrile) from Anatech (Burdick and Jackson) and formic acid (Merck South Africa) to prepare the mobile phases. Acetyl chloride and 1-butanol were obtained from Sigma-Aldrich (South Africa) and were used to prepare butanolic-HCl for the derivatisation of acylcarnitines. Acylcarnitine standards were purchased from Cerilliant (Sigma-Aldrich) and were used to create external calibration curves. The composition of each acylcarnitine mix was as follows: Acylcarnitine mix 1 [ L-carnitine (C0) and acetyl-L-carnitine (C2)] and acylcarnitine mix 2 [butyryl-L-carnitine (C4), isobutryryl-L-carnitine (iC4), isovaleryl-L-carnitine (iC5), valeryl-L-carnitine (C5), hexanoyl-L-carnitine (C6), octanoyl-L-carnitine (C8), decanoyl-L-carnitine (C10), dodecanoyl-L-carnitine (C12), tetradecoloyl-L-carnitine (C14) and octadecanoyl-L-carnitine (C18)]. Deuterated acylcarnitines were obtained from the Centre for Human Metabolomics (North-West University) and served as internal standards [methyl-d3-L-carnitine (d3-C0), d3-acetylcarnitine (d3-C2), d3-propionylcarnitine (d3-C3), d3-butyrylcarnitine (d3-C4), d9-isovalerylcarnitine (d9-iC5), d3-octanoylcarinitine (d3-C8), d3-decanoylcarnitine (d3-C10), d3-dodecanoylcarnitine (d3-C12), d3-tetradecanoylcarnitine (d3-C14), d3-octadecanoylcarnitine (d3-C18)].

### CSF optimisation and sample preparation

The analytical method was optimised for the CSF sample matrix prior to sample preparation. Known concentrations of acylcarnitine standards were spiked into blank CSF to generate external calibrators and correct for matrix effects. Pooled quality control (PQC) samples were prepared by combining aliquots from individual CSF samples into a single tube, which was aliquoted into vials and analysed with each batch. Sample preparation was carried out as described previously (Plaatjie et al., 2025). Briefly, CSF samples, external calibrators and PQC were thawed to room temperature and homogenised by vortex mixing. Extraction was achieved by adding 200 µL of internal standard (containing deuterated acylcarnitines) to 10 µL of CSF samples; the same procedure was applied to calibrators and PQC. The mixtures were vortexed and centrifuged at 12,000 x *g* at 10 °C for 10 min. A 200 µL volume of the sample supernatant was transferred to clean Eppendorf tubes and evaporated to dryness under a gentle stream of nitrogen for 1 h. The samples were derivatised by adding 100 µL of N-butanol: acetyl chloride (25:6.25, v/v) to the dried residue and incubated for 45 min at 65 °C. The butylated samples were then evaporated to dryness under a stream of nitrogen gas. The dried residue was then reconstituted with 100 µL of freshly prepared mobile phase [acetonitrile: water (50/50) (v/v), with 0.1% formic acid]. The samples were transferred to LC vials for analysis.

### LC-MS/MS

The LC-MS/MS was carried out as previously described by Plaatjie et al. (2025). Acylcarnitines were analysed using high-performance liquid chromatography coupled to a triple quadrupole tandem mass spectrometer for detection and quantification. The chromatographic separation of acylcarnitines was achieved by using a C18 Zorbax reverse phase column (2.1 mm × 100 mm × 1.8 µm) fitted with a Phenomenex guard column (2.0 µm depth filter × 0.004in ID). The column temperature was kept at 30 °C during the entire run. The mobile phases consisted of water with 0.1% formic acid (A) and acetonitrile with 0.1% formic acid (B). The elution gradient started with 5% B, followed by a linear gradient to 100% B. The flow rate was set at 0.3 mL/min for the first 13 min and increased to 0.35 mL/min for the rest of the run. The total run time for each sample was 30 min. The samples were delivered to the QQQ-MS via electrospray ionisation (ESI) in positive mode with the following source conditions: nitrogen drying gas temperature of 300 °C and a flow rate of 7.5 L/min for the remainder of the run. The compounds were analysed in multiple reaction monitoring (MRM) mode using enhanced sensitivity with the multiplier voltage set at 300 Delta EMV and a dwell time of 45 milliseconds for all metabolites. Data acquisition was achieved using Agilent’s Masshunter Workstation Software (v B02.01) and Masshunter Optimizer software (v B02.01).

### Data processing and statistical analysis

Data was extracted using Agilent’s Masshunter Workstation software (v B06.00; Qualitative Analysis and Quantitative Analysis). Samples and metabolites were individually inspected, aligned with their respective internal standards, and integrated manually. The extracted metabolite responses were then exported to an Excel file for subsequent quantification. Blank subtraction was done before quantification to account for endogenous background signal contributions from the pooled CSF. The blank-corrected calibrators were then normalised by their corresponding internal standard response. An external calibration curve was generated for each acylcarnitine metabolite using the normalised response ratios plotted against the known standard concentrations. Absolute concentrations in the samples were determined by interpolating their normalised response ratios onto the respective calibration curves. The data were summarised using the mean, median, standard deviation (SD), and interquartile range (IQR).

## Results

### Baseline characteristics

A comparison analysis of acylcarnitine profiles across TBM, VM, and NMC groups of this cohort has been reported (Plaatjie et al., 2025). For this study, only the control group was used. The following clinical characteristics were mostly present upon presentation: fever (60%), vomiting (47%), diarrhoea (21%), weight loss (14%), headache (30%), seizures (37%), cough (28%), raised intracranial pressure (2%), meningeal irritation (14%), and decreased level of consciousness (19%). The main exclusion criterion was HIV-positive or HIV-unknown status. Despite presenting with clinical symptoms of disease, all control participants were ruled out for meningitis, and their CSF parameters, including glucose, protein, and leucocyte levels, remained within normal ranges (Table 1).

**TABLE 1:** Baseline characteristics of paediatric cases.

		Paediatric cases (n = 57)
Age in months [median (IQR)]		45 (12–72)
Gender, n (%)	Female	20 (35)
	Male	37 (65)
Cerebrospinal fluid features [median (IQR)]	Normal values (Jerrard et al., 2001; Tan et al., 2023)	
Glucose (mM)	2.8–4.4	3.82 (3.4–4.3)
Protein (g/L)	0.15–0.6	0.3 (0.1–0.3)
Leucocytes (cells/µL)	0–5	1.45 (0–1.5)

### Coefficient of variance (CV)

**FIGURE 1:**
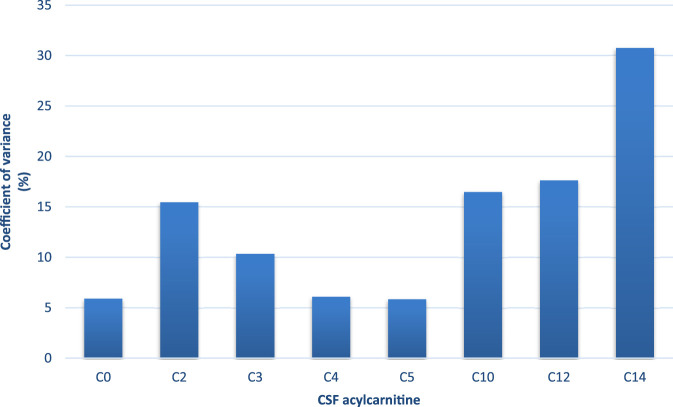
Coefficient of variance (CV; %) of each acylcarnitine calculated from the mean values of the pooled quality control samples across batches. C0-C12 had <20% CV (C0, C3, and C5 <10% CV), indicating low analytical variation. C14 had 31% CV, but this is because of the very low concentrations of this acylcarnitine.

**FIGURE 2:**
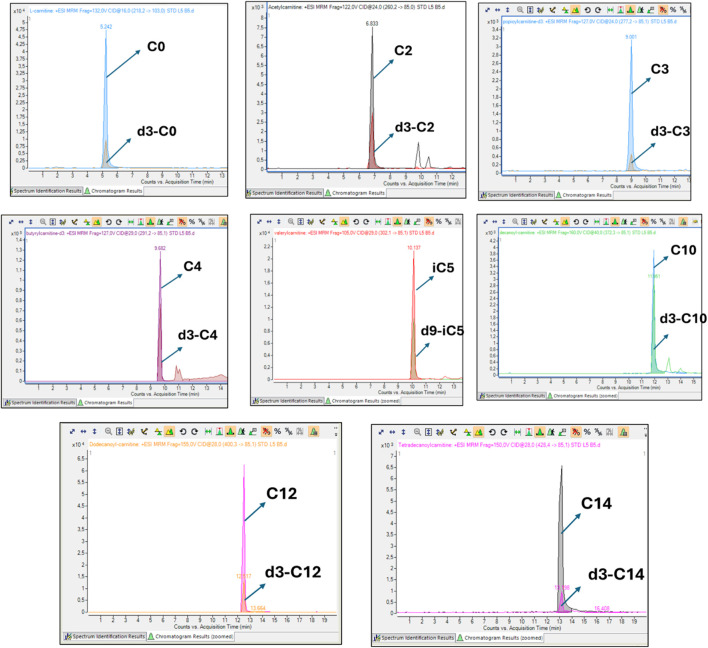
Representative overlaid chromatograms of each acylcarnitine and its corresponding isotopically labelled internal standard. In each case, the analyte and its internal standard co-elute at identical retention times, producing overlapping peaks. This demonstrates correct assignment of the metabolite, confirms that the internal standard underwent the same chromatographic and ionisation conditions as the analyte, and ensures accurate and reliable absolute quantification.

To ensure analytical reliability, pooled quality control samples were analysed together with the samples. The CV for each acylcarnitine was calculated from the mean values of the pooled QC samples across batches. Free carnitine (C0), propionylcarnitine (C3), and butyrylcarnitine (C4) exhibited the lowest CVs (<10%) while tetradecanoylcarnitine showed a slightly higher CV (31%) (Figure 1). All acylcarnitines were analysed together within each sample; thus, the higher CV observed for certain acylcarnitines may not be solely attributed to analytical variability but could also indicate the inherent instability of these metabolites. Furthermore, CSF’s naturally low acylcarnitine concentration may contribute to the elevated CV values (Bogumil et al., 2008). Figure 2 shows that the assignment of the acylcarnitines was confirmed in the LC chromatograms using deuterated internal standards of acylcarnitines. The retention times are represented by the metabolite peaks. The retention times increase with acylcarnitine chain length, demonstrating good chromatographic separation.

### CSF concentrations of acylcarnitines

**FIGURE 3:**
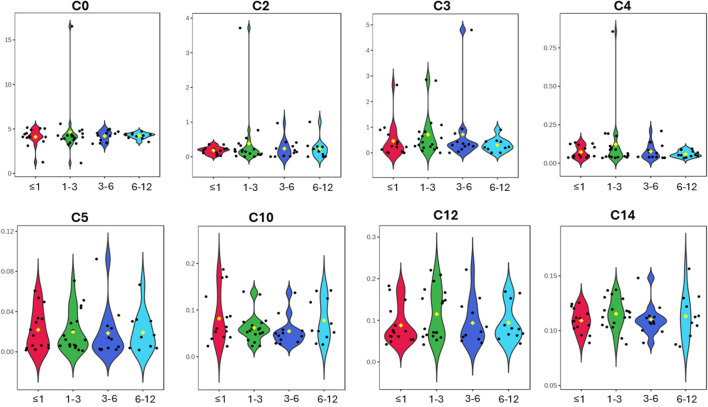
Violin plots of the eight CSF acylcarnitines measured across the four age groups of ≤1 year old, >1 and ≤3 years old, >3 and ≤6 years old, and >6 and ≤12 years old (x-axis). Concentrations on the y-axis are reported as micromoles per L (µM). No major differences in acylcarnitines are observable across the four paediatric age groups.

In this study, we report the mean, median, standard deviation, and interquartile range (IQR) concentrations of CSF acylcarnitine based on 57 samples from children (Table 2) and categorised into four age groups: ≤1 year old, >1 and ≤3 years old, >3 and ≤6 years old, and >6 and ≤12 years old (Table 3; Figure 3). Table 3 and Figure 3 show that acylcarnitine concentrations do not differ much across the paediatric age groups. Additionally, we compare these reference ranges to the CSF acylcarnitine values from adults reported in the literature (Table 2). Mandal et al. (2012) provided the mean concentration values from seven non-meningitis adults (aged 18–40 years). Carlsson et al. (2020) documented mean CSF acylcarnitine concentrations from 12 healthy adults. Plewa et al. (2019) reported the mean concentration of CSF acetylcarnitine from 10 individuals without nervous system disorders, but the age group was unspecified.

**TABLE 2:** Reference ranges of acylcarnitine concentrations determined in paediatric CSF in this study compared to literature values in adults. All concentrations reported as micromoles per L (µM).

Acylcarnitines	Children (≤12 years old) [n = 57]				Literature values (adults)		
	Mean	Median	SD	IQR	Mandal et al. (2012) Mean (±SD)	Carlsson et al. (2020) Mean (±SD)	Plewa et al. (2019) Mean (±SD)
Free carnitine (C0)	4.33	4.21	1.81	3.92–4.52	1.9 (±0.5)	1.69 (±0.263)	-
Acetylcarnitine (C2)	0.27	0.19	0.51	0.07–0.29	0.322 (±0.148)	0.407 (±0.158)	0.457 (±0.208)
Propionylcarnitine (C3)	0.65	0.34	0.87	0.23–0.75	0.018 (±0.009)	-	-
Butyrylcarnitine (C4)	0.09	0.05	0.11	0.04–0.1	0.024 (±0.007)	0.136 (±0.003)	-
Valerylcarnitine (C5)	0.02	0.01	0.02	0.004–0.03	0.013 (±0.006)	0.079 (±0.003)	-
Decanoylcarnitine (C10)	0.07	0.06	0.04	0.04–0.08	-	-	-
Dodecanoylcarnitine (C12)	0.1	0.07	0.05	0.06–0.15	-	-	-
Tetradecanoylcarnitine (C14)	0.11	0.11	0.02	0.1–0.12	-	0.058 (±0.005)	-

IQR, interquartile range; SD, standard deviation.

**TABLE 3:** Reference ranges of acylcarnitine concentrations determined in paediatric CSF in this study according to four age groups: ≤1 year old, >1 and ≤3 years old, >3 and ≤6 years old, and >6 and ≤12 years old. All concentrations reported as micromoles per L (µM).

		Free carnitine (C0)	Acetylcarnitine (C2)	Propionylcarnitine (C3)	Butyrylcarnitine (C4)	Valerylcarnitine (C5)	Decanoylcarnitine (C10)	Dodecanoylcarnitine (C12)	Tetradecanoylcarnitine (C14)
Children (≤1 year old) [n = 15]	Mean	4.11	0.18	0.58	0.07	0.02	0.08	0.09	0.11
	Median	4.11	0.19	0.31	0.05	0.02	0.06	0.07	0.11
	SD	0.97	0.1	0.73	0.04	0.02	0.06	0.05	0.01
	IQR	4.05–4.70	0.11–0.23	0.21–0.76	0.04–0.11	0.01–0.03	0.04–0.11	0.06–0.11	0.1–0.12
Children (>1 and ≤3 years old) [n = 19]	Mean	4.65	0.41	0.56	0.1	0.02	0.02	0.06	0.01
	Median	4.17	0.15	0.48	0.06	0.01	0.06	0.08	0.12
	SD	3.01	0.83	0.82	0.19	0.02	0.03	0.06	0.01
	IQR	3.81–4.43	0.08–0.27	0.25–0.82	0.04–0.11	0.01–0.03	0.05–0.07	0.06–0.16	0.11–0.12
Children (>3 and ≤6 years old) [n = 12]	Mean	4.21	0.24	0.78	0.08	0.02	0.06	0.09	0.11
	Median	4.22	0.15	0.43	0.07	0.01	0.05	0.07	0.11
	SD	0.59	0.28	1.36	0.05	0.03	0.03	0.05	0.01
	IQR	3.67–4.57	0.02–0.33	0.26–0.6	0.04–0.09	0.003–0.02	0.04–0.06	0.06–0.12	0.105–0.11
Children (>6 and ≤12 years old) [n = 11]	Mean	4.25	0.24	0.37	0.06	0.02	0.08	0.09	0.11
	Median	4.34	0.21	0.31	0.05	0.02	0.06	0.08	0.11
	SD	0.33	0.28	0.23	0.02	0.02	0.05	0.05	0.02
	IQR	4.09–4.42	0.04–0.29	0.21–0.45	0.05–0.07	0.005–0.03	0.05–0.12	0.07–0.12	0.1–0.13

IQR, interquartile range; SD, standard deviation.

## Conclusion

This study is the first to characterise normal CSF acylcarnitine concentrations in children without a brain infection (i.e., with little to no CSF leucocytes), providing a critical reference for future research. The slight differences observed in paediatric cases compared to adult studies highlight the impact of sample size and age on CSF composition, underscoring the need for more paediatric-focused metabolomics research. These baseline values serve as a foundation for identifying disease-associated metabolic alterations in neurological disorders and improving diagnostic approaches in paediatric populations.

## Data Availability

The raw data supporting the conclusions of this article will be made available by the authors, without undue reservation.
